# Preparation and Material–Structure–Performance Relationships of Biaxially Stretched Polytetrafluoroethylene (PTFE) Membranes for Air Filtration

**DOI:** 10.3390/polym18020199

**Published:** 2026-01-11

**Authors:** Chunxing Zhou, Haiqin Mo, Yiqin Shao, Parpiev Khabibulla, Juramirza Abdiramatovich Kayumov, Guocheng Zhu

**Affiliations:** 1 College of Textile Science and Engineering, Zhejiang Sci-Tech University, Hangzhou 310018, China; 2 Department of Technology of Goods of Textile Industry, Faculty of Textile Engineering, Namangan State Technical University, 12 Karimov Street, Yangi Namangan District, Namangan City 160100, Uzbekistan; 3 Department of Mechanical Engineering, Faculty of Construction, Samarkand State Architecture and Construction University, Samarkand 140143, Uzbekistan

**Keywords:** biaxial stretching, PTFE membrane, air filtration, structure–property relationship, air permeance

## Abstract

Biaxially stretched polytetrafluoroethylene (PTFE) membranes are promising media for high-efficiency air filtration because of their stable node–fiber microstructure and environmental durability. To clarify how resin properties and microstructure govern filtration behavior, ten PTFE resins with different average molecular weights (Mn) and particle size characteristics were processed into membranes under essentially identical biaxial stretching and sintering conditions. Resin particle size, fiber diameter and pore size distributions were quantified, and coefficients of variation (CVs), together with Spearman rank correlations, were used to analyze material–structure–performance links. Filtration efficiency, pressure drop and quality factor (QF) were measured according to ISO 29463-3 using 0.1–0.3 μm aerosols. Higher Mn combined with lower particle-size dispersion favored finer fibers and narrower pores, yielding efficiencies close to 100%, but increased pressure drop and slightly reduced QF, indicating a trade-off between efficiency and flow resistance. The sample with the lowest Mn in its group and a high machine-direction draw ratio (12×), showed pronounced fibril breakage, node coalescence, broadened pore-size distribution and degraded QF, illustrating the sensitivity of structure and performance to resin-process mismatch. Overall, the study establishes a hierarchical material–fiber–pore–performance relationship that can guide resin selection, structural tuning and process optimization of biaxially stretched PTFE membranes.

## 1. Introduction

Air pollution is one of the most pressing environmental and public health challenges worldwide. Rapid industrialization, urbanization and increased energy consumption have led to a sharp rise in the emission of fine particulate matter (PM_2.5_ and PM_10_), which poses severe threats to human health and the ecological environment. The World Health Organization (WHO) reports that exposure to PM_2.5_ and related air pollutants causes millions of premature deaths each year [1,2], and nearly 99% of the global population now lives in areas where PM_2.5_ concentrations exceed WHO guideline limits [3,4,5]. Therefore, a large body of evidence indicates that long-term exposure to PM_2.5_ is closely associated with reduced life expectancy and a substantial mortality burden [3,5,6]. Moreover, epidemiological studies have reported excess mortality risks even at relatively low ambient PM_2.5_ levels, suggesting that PM_2.5_ exposure may not have an absolute “safe threshold” [7].

In this context, high-efficiency particulate air (HEPA) filters based on borosilicate glass microfibers, melt-blown polypropylene (PP) electret nonwovens, electrospun nanofiber webs and membrane–fiber laminates are widely used [8]. The performance classification and testing of these filter media are governed by rigorous standards. For example, ISO 29463 and EN 1822 specify the classification and performance grades of EPA/HEPA/ULPA filters [9,10], while relevant Chinese standards—such as GB 2626-2019 for respiratory protection and GB/T 14295-2019 for air filters—also define corresponding test methods and requirements [11,12]. Meanwhile, aerosol characterization and calibration strategies for ensuring reliable filtration testing have been systematically summarized in standards and review literature [13]. However, these conventional media often suffer from brittleness, limited reusability and end-of-life recyclability, poor chemical resistance and high airflow resistance at the most penetrating particle size MPPS, ≈0.1–0.3 μm [8,14]. PP electret filters rely on stored electrostatic charges that decay under humid or thermal conditions [15,16,17], while electrospun nanofiber filters, though highly efficient, generally lack sufficient mechanical robustness and environmental durability [18,19,20]. In addition, previous studies have shown that decontamination or reuse treatments for electret-based masks often lead to a noticeable deterioration in filtration performance, further highlighting the limitations of such media in repeated-use scenarios [21]. These limitations motivate the search for alternative filter materials with stable, charge-independent and reusable performance. Because most current HEPA media are based on relatively low-cost polymers such as PP, any alternative material—such as PTFE—must therefore justify its higher raw-material and processing costs by offering clearly superior durability and service lifetime [14,22,23].

PTFE microporous membranes offer a distinct approach, relying on a physical filtration mechanism governed by microstructure rather than surface charge. Structurally, they consist of an interconnected node–fiber network, where nanoscale fibrils bridge crystalline nodes to form a pore structure with high specific surface area and a controllable pore-size distribution [14,22]. This architecture provides high capture efficiency, low pressure drop and good mechanical resilience: the node–fiber skeleton resists collapse or compaction under high humidity or temperature, preserving porosity and permeability over extended use [19,22]. The purely mechanical filtration mechanism avoids charge dissipation issues that limit the lifespan of electret and electrospun media [15,16,17], while strong C-F bonds afford excellent chemical inertness against acids, solvents and oxidizing gases [22,23]. Consequently, PTFE membranes have been reported to be repeatedly cleaned and reused with minimal loss of performance, making them attractive for high-performance air filtration [14,22]. Previous studies have shown that PTFE hollow-fiber filter media can retain nearly unchanged filtration efficiency after cleaning following fouling, demonstrating favorable reusability [22]. This cleanability and reusability make PTFE membranes highly promising for high-performance air filtration applications [14,23]. Although PTFE is more expensive than conventional filter polymers such as PP, its outstanding environmental durability and potential for repeated cleaning and reuse can offset the higher initial material cost in long-term or harsh-environment applications [14,22,23]. To further enhance performance and tailor microstructure, biaxial stretching has become one of the key processing routes for PTFE membranes [14,24]. In practice, sequential stretching in the machine and transverse directions, followed by sintering, induces fibrillation of PTFE crystallites and develops the characteristic node–fiber architecture [25,26,27].

The filtration behavior of biaxially stretched PTFE membranes is highly sensitive to the coupled effects of multiple processing parameters, such as stretch ratio, temperature, strain rate and sintering conditions, which together determine membrane porosity, pore size and fiber diameter [14,28]. Increasing stretch ratio generally promotes higher porosity and smaller mean pore size, thereby improving filtration efficiency, but excessive stretching may compromise mechanical integrity [24,28]. Processing temperature and strain rate further influence molecular orientation, fiber uniformity and defect density, while overly aggressive sintering can lead to structural coarsening and reduced pore stability [25,28,29]. In addition, the characteristics of the raw resin—including particle size, morphology and crystallinity—strongly affect the fibrillation process and the resulting pore architecture [14,19]. Although many studies have examined single factors such as stretch ratio or sintering temperature, there is still a lack of systematic investigations into the coupled effects of resin properties and stretching parameters, as well as quantitative links between microstructural descriptors (e.g., fiber diameter and pore structure) and key filtration metrics such as efficiency, pressure drop, dust-holding capacity and cleanability [27,28]. Therefore, a unified quantitative framework that links multiscale structural descriptors of the membrane (e.g., fiber diameter and pore size distribution) to key performance metrics (e.g., filtration efficiency and pressure drop) has not yet been established [14].

Against this background, a deeper understanding of how resin properties and stretching paths jointly determine the multiscale structure and filtration behavior of PTFE membranes is of significant scientific and engineering interest. Clarifying the roles of resin molecular weight and particle size characteristics in the formation of the node–fiber network, and quantitatively elucidating the relationships between fiber diameter and pore-size distributions and filtration efficiency, pressure drop and QF, can provide a rational basis for resin selection and process design. In particular, introducing quantitative descriptors such as the CV for particle, fiber and pore size distributions, together with Spearman rank correlation analysis to capture monotonic trends between material parameters, structural metrics and performance indicators, enables a more rigorous evaluation of the hierarchical linkage from resin properties to membrane microstructure and filtration behavior. Compared with previous studies that mainly focused on single processing parameters, the present work aims to simultaneously account for differences in resin characteristics and stretching conditions, and to establish a unified analytical framework that connects resin parameters, fiber-scale morphology and pore structure to filtration performance.

In this study, ten types of PTFE resins are used to prepare biaxially stretched PTFE membranes via controlled multi-stage stretching and sintering. The particle-size characteristics of the resins, as well as the node–fiber morphology, fiber-diameter distributions and pore-size distributions of the membranes, are systematically characterized, and filtration performance is evaluated under representative aerosol challenge conditions in terms of filtration efficiency, pressure drop and QF. Strategically, the ten types of PTFE resins are divided into several stretching-process groups: within each group, stretching temperature, draw ratio, stretching speed and sintering schedule are kept essentially constant, while resin molecular weight and particle size characteristics are varied to examine the influence of material parameters on structure and performance; comparisons between different process groups then reveal how changes in key processing parameters further modulate the material–structure–performance relationships. By establishing quantitative correlations between resin parameters, microstructural features and filtration metrics within this design—fixing the process to compare materials within groups and comparing processes across groups—this work seeks to elucidate the material–structure–performance relationships of biaxially stretched PTFE membranes and to provide guidance for resin selection and process-window optimization for durable, high-efficiency PTFE air filtration media.

## 2. Materials and Methods

### 2.1. Materials

Ten types of PTFE resins are offered by Zhejiang Zhaohui Filtration Technology Co., Tongxiang City, China. The sample information is shown in Table 1. The additive oil is isoalkanes (flash point 97 °C, density 790 kg/m^3^, viscosity 2.133 mPa·s, molecular weight 188 g/mol) was supplied by ExxonMobil Chemical Business (Shanghai) Co., Shanghai, China. In addition, a commercial PP electret filter was also provided by Zhejiang Zhaohui Filtration Technology Co., with a basis weight of 20 g/m^2^ and a thickness of approximately 125 ± 5.37 μm, and was used as a benchmark material for comparison.

### 2.2. Fabrication of Biaxially Stretched PTFE Membranes

**Figure 1:**
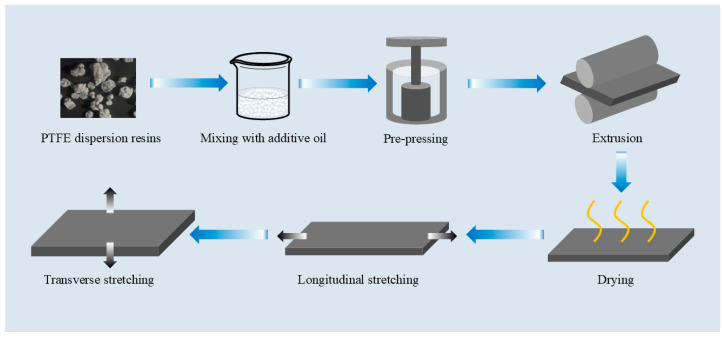
Preparation process of biaxially stretched PTFE membrane. Black arrows denote the stretching direction, whereas blue arrows indicate the process flow (i.e., the sequence of steps).

The preparation process of the biaxially stretched PTFE membranes is shown in Figure 1. First, the PTFE powder was stored in a cold room at a temperature below 19 °C for 24 h to ensure a fully cooled, loose and uniform initial state of the powder, reduce agglomeration, and provide consistent raw-material conditions for subsequent mixing and paste extrusion. Subsequently, the additive oil was added to the resin powder according to the process parameters listed in Table 1, and the mixture was sealed and stirred for 90 min to ensure thorough mixing between the PTFE resin and the additive oil. The mixture was then matured in an oven at 45 °C for 16 h. After maturation, the material was pre-pressed and molded: the conditioned mixture was loaded into a cylindrical mold, the pre-pressing pressure was set to 2.5 MPa, and the pre-pressing time was 20 min, yielding a dense cylindrical billet. This billet was then slowly extruded through a die to obtain a cylindrical extrudate, which was passed through a water-bath calender to form a base tape with a thickness of approximately 1.8–2.0 mm and a length of about 2 m. The base tape was dried to remove the additive oil and then fed into a longitudinal stretching machine to obtain a longitudinally stretched film by applying a suitable stretch ratio according to the production process. Subsequently, the longitudinally stretched film was transferred to a transverse stretching machine, where the transverse stretching speed, upper clamping distance, preheating temperature and a three-stage sintering profile (320, 350 and 260 °C) were set according to the process requirements of the different PTFE resins. Specifically, the film was heated from room temperature to 320 °C at a rate of approximately 2 °C/min and held for 30 min, then further heated to 350 °C at 2 °C/min and held for 2 min, followed by controlled cooling to 260 °C at 5 °C/min and holding for 30 min before being cooled down to room temperature. Through this process sequence, biaxially stretched PTFE membranes meeting the specified performance requirements were obtained.

As shown in Table 1, the process parameters of samples 1–4, 5–7, and 8–10 are basically consistent. Therefore, in the following discussion, the 10 kinds of samples are divided into three groups for analysis: Group 1 (Samples 1–4), Group 2 (Samples 5–7), and Group 3 Samples (8–10).

### 2.3. Characterization Methods

The morphology of the PTFE resin was determined by using a Zeiss polarizing microscope (Axio Cam Erc 5S, Carl Zeiss AG, Oberkochen, Germany), and the particle size of the resins was analyzed using Nano Measure software (image–Pro Plus) The surface morphology of the biaxially stretched PTFE membrane was determined with a field emission scanning electron microscope (FESEM, Gemini500, Carl Zeiss AG, Oberkochen, Germany), and the fiber diameters were analyzed using Nano Measure software. The thickness of the biaxially stretched PTFE membranes was measured using a digital thickness gauge (BK-3281, Shanghai Dingleng Industrial Development Co., Shanghai, China) at 5 positions and averaged. The pore size distribution of the biaxially stretched PTFE membrane was determined using the bubble point method with a capillary flow porometer (Porometer Porolux 500, Aptco Technologies NV, Nazareth, Belgium), with Galpore as the wetting liquid. The obtained pore size represents an equivalent hydraulic constriction of the interconnected through-pores (i.e., flow-based pore size) rather than a sieve-like geometric aperture. The air permeance of the biaxially stretched PTFE membranes was measured using an automatic digital fabric air permeability tester (YG461E-II, Wuhan Guoliang Instrument Co., Wuhan, China) at a pressure differential of 200 Pa and a test area of 20 cm^2^, and the same conditions were applied to the PP electret filter for comparison.

### 2.4. Filtration Performance Evaluation

With reference to ISO 29463-3:2011 “High-efficiency filters and filter media for removing particles in air-Part 3: Testing flat sheet filter media” [30], the filtration performance of the biaxially stretched PTFE membranes was evaluated using an automatic filter media tester with an effective test area of 100 cm^2^. During testing, each PTFE membrane was placed in a flat-sheet configuration over the test area, and a very thin, highly porous nonwoven fabric was attached to the backside as a mechanical support to prevent rupture of the stretched film under clamping and loading; the structure of this backing layer was extremely sparse, so its influence on the measured filtration efficiency and pressure drop was negligible [31]. During the preliminary tests, ten types of PTFE membrane were first evaluated under identical conditions using NaCl aerosol with a particle size of 0.3 μm. The results showed that Samples 5–10 exhibited very high filtration efficiencies that were also extremely close to each other, making it difficult to distinguish their performance. To improve the resolution of performance differences within this group, more stringent test conditions were adopted for Samples 5–10 in the subsequent formal measurements, namely paraffin oil aerosol with a particle size of 0.1–0.2 μm and an increased face velocity. Combined with the pore-size characterization results, the final operating conditions for filtration performance evaluation were set as follows: for Samples 1–4, the face velocity was 5.3 cm/s and the test aerosol was NaCl with a particle size of 0.3 μm, whereas for Samples 5–10, the face velocity was 15 cm/s and the test aerosol was paraffin oil with a particle size of 0.1–0.2 μm.

## 3. Results and Discussion

### 3.1. Molecular-Weight, Particle-Size and Thickness Characteristics of PTFE Resins and Membranes

The Mn of the PTFE resins is listed in Table 1. Group 1 (Samples 1–4) exhibits Mn ranges from 1.13 × 10^7^ to 4.92 × 10^7^ g·mol^−1^; Group 2 (Samples 5–7) covers a wider range, from 2.81 × 10^7^ to 1.19 × 10^8^ g·mol^−1^; Group 3 (Samples 8–10) lies in the intermediate range of 3.94 × 10^7^ to 6.81 × 10^7^ g·mol^−1^. Overall, the molecular weights of the resins used in this work span nearly one order of magnitude. Since Mn reflects chain length and entanglement density, higher Mn is favorable for forming a continuous and stable node–fiber network and for reducing the risk of necking and fracture. Notably, Sample 4 has the lowest Mn (1.13 × 10^7^ g·mol^−1^), indicating shorter polymer chains and lower entanglement density, which will later be linked to its abnormal structural behavior.

**Figure 2:**
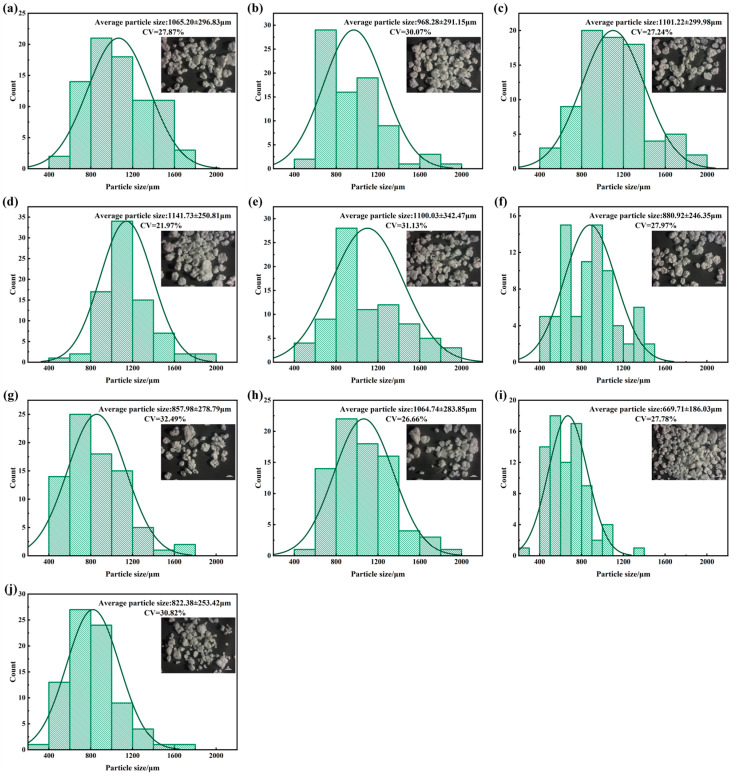
Morphology (×10) and particle size distributions of PTFE resin: (**a**–**j**) correspond to Samples 1–10, respectively. Particle size analysis was obtained by measuring 80 resin particles across six randomly selected fields of view.

Figure 2 presents the morphology and particle size distributions for Group 1 (Samples 1–4), Group 2 (Samples 5–7) and Group 3 (Samples 8–10), respectively. Group 1 exhibits the largest average particle size ranges from 968.25 to 1141.75 µm, centered around approximately 1.0 mm; Group 2 shows slightly smaller average particle sizes, ranging from 857.98 to 1100.03 μm. Group 3 falls in an overall smaller interval, ranging from 669.71 to 1064.74 μm, indicating that Group 3 is marginally smaller than Group 2. In general, all resin samples show approximately spherical shapes with smooth surfaces and slight agglomeration, while Group 3 exhibits more complex and diverse local morphologies.

To compare distribution breadth under different means, we adopt CV as a normalized dispersion metric, according to Equation (1):

(1) $$CV=\frac{\sigma }{\mu }\times 100\%$$

where σ denotes the standard deviation and μ the mean. Unlike reporting σ alone, CV enables direct comparison of uniformity across datasets with different magnitudes. For resin particle size, a lower CV indicates a more uniform size distribution, which typically facilitates more homogeneous dispersion during processing and more consistent fibrillation; in contrast, resins with larger mean size and/or irregular morphology may lead to non-uniform fiber formation.

As show in Figure 2, narrower and more symmetric particle size distributions typically correspond to lower CV, and the associated micrographs exhibit particle ensembles with more uniform sizes and relatively compact local structure. In contrast, broader distributions or those with a slight “tail” tend to correspond to higher CV, accompanied by more pronounced agglomeration and size heterogeneity. These findings lay a foundation for establishing the material–fiber–pore structure relationships discussed in the subsequent sections.

In addition, Table 1 summarizes the membrane thickness before and after biaxial stretching. The pre-stretched tapes have thicknesses of approximately 260–340 μm, with only moderate variations among samples, reflecting the comparable paste-extrusion and calendering conditions used during resin pre-processing. After biaxial stretching and sintering, the final membrane thickness is reduced to about 3.4–5.8 μm, corresponding to a thickness reduction factor on the order of 60–90. Within each process group, the post-stretched thicknesses are very similar, indicating that the applied draw ratios and stretching temperatures primarily control the overall densification, whereas differences in Mn and particle-size characteristics mainly manifest in the finer details of fiber diameter and pore structure.

### 3.2. Evolution of Fiber Morphology and Fiber Diameter Distribution of Biaxially Stretched PTFE Membranes

**Figure 3:**
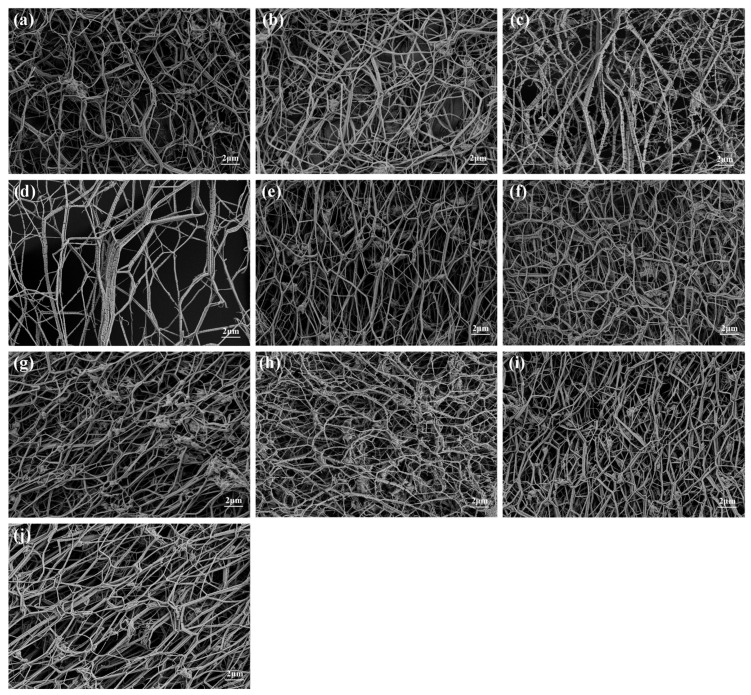
SEM images (×5000, insets): (**a**–**j**) correspond to Samples 1–10, respectively.

**Figure 4:**
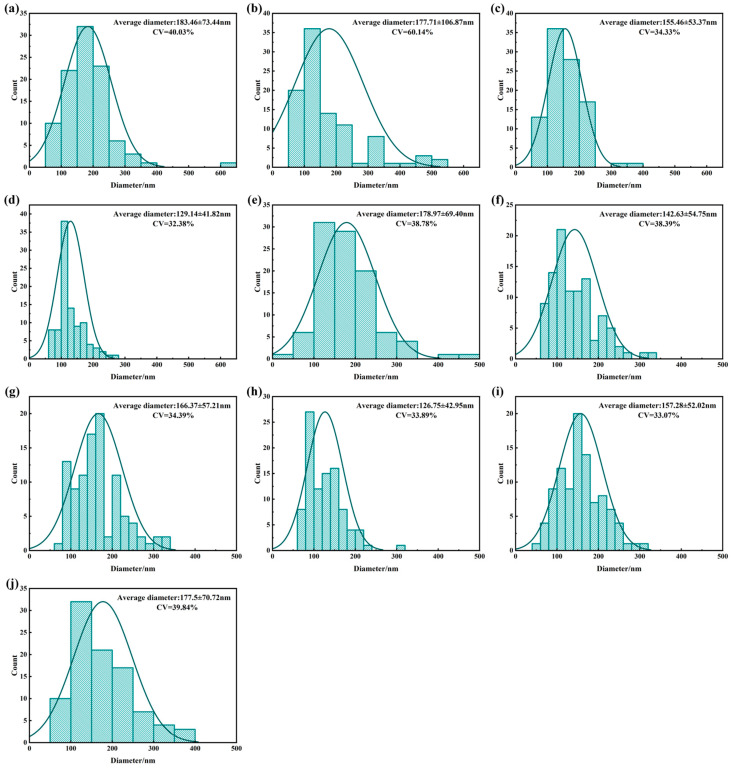
Fiber diameter distributions of biaxially stretched PTFE membranes: (**a**–**j**) correspond to Samples 1–10, respectively. Fiber diameter analysis was obtained by measuring about 95 fiber diameters across six randomly selected fields of view.

Figure 3 and Figure 4 present the surface morphology and fiber diameter distributions of the PTFE membranes for Group 1 (Samples 1–4), Group 2 (Samples 5–7), and Group 3 (Samples 8–10), respectively. From the image comparison, Group 1 exhibits coarse fiber networks with frequent node merging and irregular pores, resulting in the broadest diameter distribution. Group 2 presents smoother and more continuous fibers with better alignment and narrower diameter spread. Group 3 features the finest and most uniformly distributed fibers, corresponding to the lowest CV. These trends indicate a clear evolution in fiber morphology from coarser and less uniform (Group 1) to finer and more consistent structures (Group 3). The observed changes in CV are consistent with SEM-based spatial uniformity and earlier CV analysis of resin particle size.

**Figure 5:**
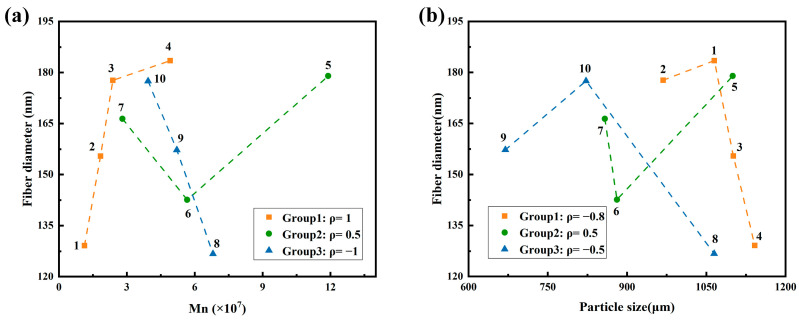
Relationships between material parameters and fiber diameter: (**a**) Mn and fiber diameter for Samples 1–10; (**b**) Resin particle size and fiber diameter for samples 1–10. The legend reports the within-group Spearman rank correlation coefficient (*ρ*).

Figure 5a,b summarize the relationships between Mn and fiber diameter, and between resin particle size and fiber diameter. To assess monotonic trends within each group, the Spearman rank correlation coefficient (*ρ*) was used. It is defined as the Pearson correlation of rank-transformed variables, which is given in Equation (2):

(2) $$\rho =corr(rank\left(X\right),rank(Y))$$

In the absence of ties, the closed form is $\rho =1-\frac{6\sum d_{i}^{2}}{n(n^{2}-1)}$, where $d_{i}$ is the rank difference for the i-th pair and n is the number of paired observations within the group (Group 1: n = 4; Group 2: n = 3; Group 3: n = 3). Given the limited sample size in Groups 2 and 3, these values serve as descriptive indicators of trend rather than statistical significance.

In Figure 5a, Group 1 shows a strong positive correlation (*ρ* = 1) between Mn and fiber diameter, indicating that higher Mn leads to larger fibers. Group 2 exhibits a moderate positive correlation (*ρ* = 0.5), while Group 3 displays a strong negative correlation (*ρ* = −1), where higher Mn results in finer fibers. In Figure 5b, the correlation between resin particle size and fiber diameter is negative in Group 1 (*ρ* = −0.8), moderate and positive in Group 2 (*ρ* = 0.5), and weakly negative in Group 3 (*ρ* = −0.5).

From a materials perspective, higher Mn promotes entangled microfibril network formation, while smaller, more uniform particles improve stress transfer and fiber homogeneity. However, the variation in correlations suggests that fiber diameter is governed by the combined effect of material properties and processing. Although processing parameters were generally consistent, the 12× longitudinal draw ratio in Group 1 may have overstretched low-Mn samples (e.g., Sample 4), leading to defects. In contrast, the reduced transverse draw speed in Group 3 (3.08 m/s) likely allowed more uniform fiber development. These findings highlight the need for coordinated optimization of resin properties (Mn, particle size) and processing conditions (draw ratio, strain rate, temperature, etc.). This interaction will be further discussed in the context of pore structure and filtration performance.

In conclusion, fiber diameter is determined by a combination of molecular weight, particle features, and process parameters. While higher Mn and finer particle size generally favor thinner and more uniform fibers, their actual influence depends on careful process matching.

### 3.3. Pore Size Distributions and Fiber–Pore Structural Relationship of Biaxially Stretched PTFE Membrane

**Figure 6:**
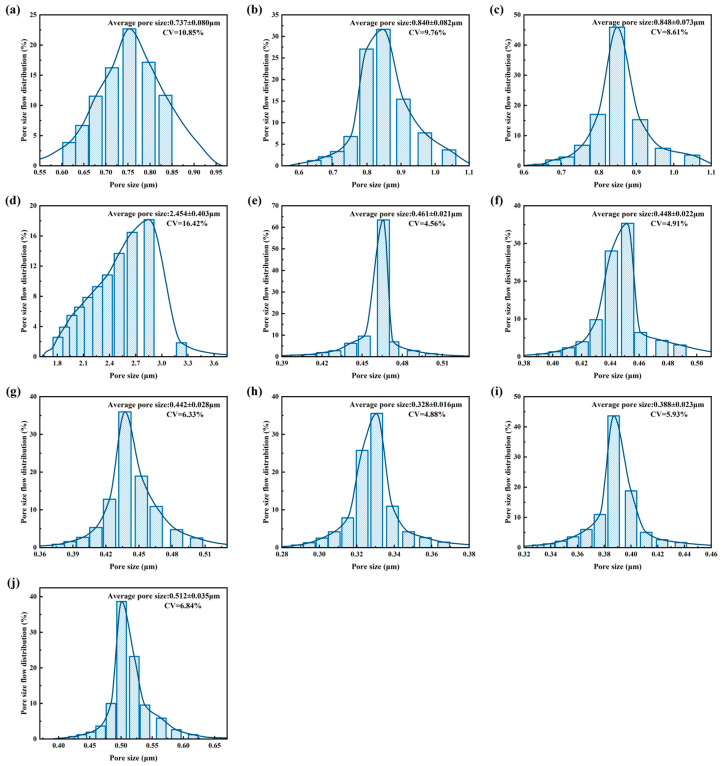
Pore size flow distribution of biaxially stretched PTFE membrane: (**a**–**j**) correspond to Samples 1–10, respectively.

The pore size flow distributions of PTFE membranes from Group 1 (Samples 1–4), Group 2 (Samples 5–7), and Group 3 (Samples 8–10) are shown in Figure 6. Group 1 exhibits a relatively large average pore size, with a broad and asymmetric histogram. In particular, Sample 4 shows a significantly larger mean pore size (2.454 μm) and higher CV compared to other samples in the same group, which is consistent with its localized node merging during fibrillation and structural instability during sintering, resulting in irregular and highly variable pore structures. In contrast, Group 2 shows a marked reduction in average pore size, with narrower and more symmetric distributions, indicating more uniform stretching and a stable mesh architecture. Group 3 further reduces average pore size and achieves the narrowest and most symmetric distribution, reflecting a denser, more uniform, and highly interconnected fibrous network.

Overall, average pore size decreases progressively from Group 1 to Group 3, accompanied by a narrowing of the pore size distribution. This trend corresponds with the convergence of fiber diameter CV discussed earlier, suggesting that improved uniformity at the fiber scale is effectively transferred to the pore network, stabilizing the structure and reducing pore size.

**Figure 7:**
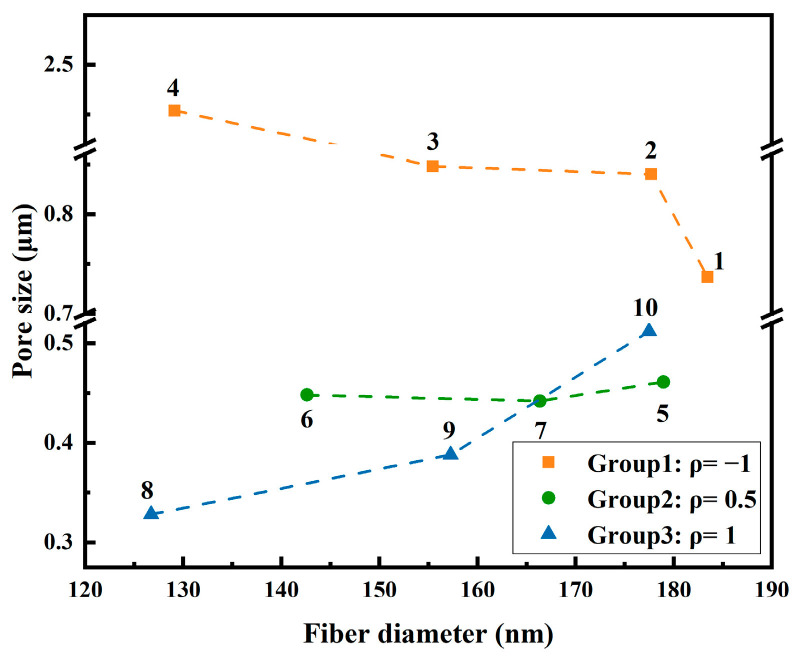
Relationship between fiber diameter and pore size for Samples 1–10. The legend reports the within-group Spearman rank correlation coefficient (*ρ*).

Figure 7 summarizes the relationship between average fiber diameter and corresponding average pore size for each group, along with the Spearman correlation coefficient *ρ* to quantify monotonic trends. In Group 1, *ρ* = −1, indicating a perfect negative monotonic relationship: as fiber diameter increases, average pore size decreases. This is consistent with the large, irregular pores and structural defects observed in Sample 4. In Group 2, *ρ* = 0.5, exhibits a moderate positive correlation, where slightly thicker fibers correspond to slightly larger pores under generally uniform network conditions. In Group 3, *ρ* = 1, shows a perfect positive monotonic trend, where the highly uniform fibrous network results in fiber diameter directly determining inter-fiber spacing and pore size. Given the limited number of samples in Groups 2 and 3, the *ρ* values are used as indicators of trend rather than definitive statistical conclusions. Importantly, these correlation trends align with changes in pore size CV across the groups.

In summary, networks formed by finer and more uniformly distributed fibers tend to produce smaller and more narrowly distributed pores, whereas coarser and more heterogeneous fibers result in larger and broader pore size distributions. Structurally, fine fibers support the formation of uniform pore networks, while coarse fibers are more likely to create looser and more variable pore frameworks. These findings demonstrate that microstructural control at the fiber scale can effectively influence the pore architecture, thereby providing a structural basis for linking pore size distribution to filtration performance in subsequent sections.

### 3.4. Filtration Performance and Pore-Structure-Performance Relationship of Biaxially Stretched PTFE Membrane

Although the bubble-point pore sizes exceed the test particle sizes, effective capture of 0.1–0.3 μm aerosols is still achieved because particle removal is governed by depth filtration via diffusion and interception rather than simple sieving.

**Figure 8:**
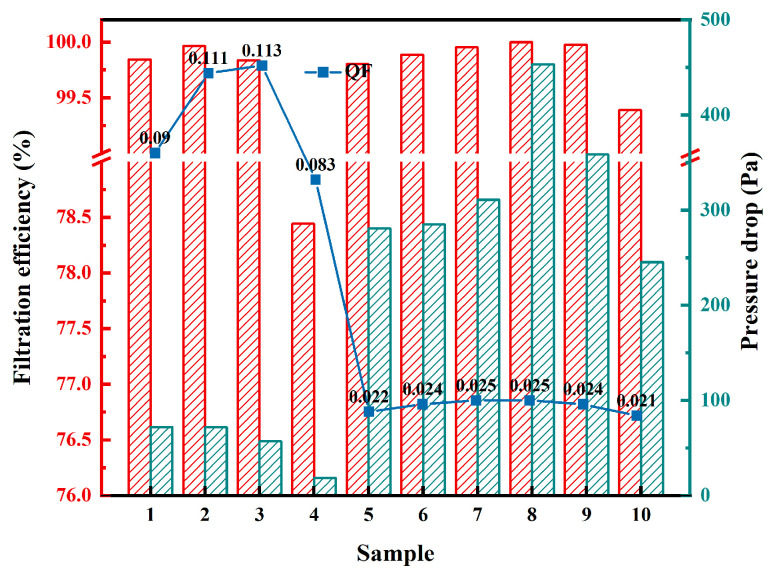
Filtration performance of biaxially stretched PTFE membrane. The red curve indicates filtration efficiency, and the green curve indicates pressure drop.

Figure 8 summarizes the filtration efficiency (η), pressure drop (ΔP), and corresponding QF for the biaxially stretched PTFE membranes prepared from 10 kinds of PTFE resins. Filtration performance was evaluated reference ISO 29463-3:2011 “High-efficiency filters and filter media for removing particles in air–Part 3: Testing flat sheet filter media” [30]. Measurements were conducted using an automated filter media tester with an effective test area of 100 cm^2^. The PTFE membranes were mounted in flat-sheet form, supported on the backside by a thin and highly porous nonwoven fabric to provide mechanical reinforcement. Given the sparsity of this support layer, its influence on efficiency and pressure drop is considered negligible [31].

In preliminary tests, ten types of PTFE membrane were first evaluated using 0.3 μm NaCl aerosol under identical conditions. Samples 5–10 already showed very high and very similar efficiencies, so more stringent conditions were adopted in the final protocol to better resolve performance differences within this group. Accordingly, and taking into account the measured pore sizes, the following conditions were used in Figure 8: for Samples 1–4, the face velocity was set to 5.3 cm/s with 0.3 μm NaCl aerosol, whereas for Samples 5–10, the face velocity was increased to 15 cm/s and the test aerosol was paraffin oil with a particle size of 0.1–0.2 μm. Because the testing conditions differ in particle size and face velocity, direct numerical comparisons across groups are not strictly meaningful; instead, the data are interpreted as qualitative references across groups, while within-group trends reflect the influence of pore structure, particularly pore size and its distribution.

The filtration efficiency η was calculated as

(3) $$\eta =\left(1-\frac{C_{down}}{C_{up}}\right)\times 100\%$$

where $C_{up}$ and $C_{down}$ are the upstream and downstream particle number concentrations, respectively.

In Figure 8, Group 1 shows the greatest performance variability. Sample 4, with significantly larger and more dispersed pores, exhibits abnormally low efficiency and pressure drop. Group 2 demonstrates generally higher efficiency and moderately increased pressure drop compared to Group 1, indicating a denser and more uniform pore network. Group 3 achieves near-100% efficiency with the highest pressure drop, consistent with its finer and more uniform pore structure.

To evaluate efficiency and resistance within a single metric, the quality factor QF is adopted, as defined by Equation (4):

(4) $$QF=\frac{-ln(1-\eta )}{∆P}$$

As indicated by the polyline in Figure 8, Group 1 exhibits the highest QF values overall, excluding Sample 4 as an outlier. Group 2 maintains high efficiency but, together with a noticeable increase in pressure drop, yields only moderate QF values. Although Group 3 reaches nearly perfect filtration efficiency, the substantially higher pressure drop results in QF values that are lower than those of Group 1 and comparable to, or slightly lower than, those of Group 2. Sample 4, due to its large pore size and reduced η, exhibits a markedly lower QF.

Given the scale difference between fiber diameter (nanometer scale) and pore size (micrometer scale), particle capture in these membranes is primarily governed by diffusion and interception mechanisms. Under the tested particle size range (0.1–0.3 μm) and airflow conditions, inertial impaction and gravitational settling play minimal roles. As pore size decreases, airflow channels narrow and local velocity increases, enhancing both diffusion-induced collisions and interception likelihood, thus significantly improving capture efficiency. Conversely, excessively large pore size may form “short-circuit” paths, allowing particles to bypass the fibrous network. Narrower pore size distributions reduce the presence of large pores, enhancing the consistency and stability of filtration efficiency. Additionally, finer fibers strengthen diffusion capture, while denser fiber networks restrict airflow pathways, enhancing interception and overall filtration performance. Overall, reducing average pore size and narrowing its distribution improves filtration efficiency but inevitably increases ΔP, thereby reducing QF.

**Figure 9:**
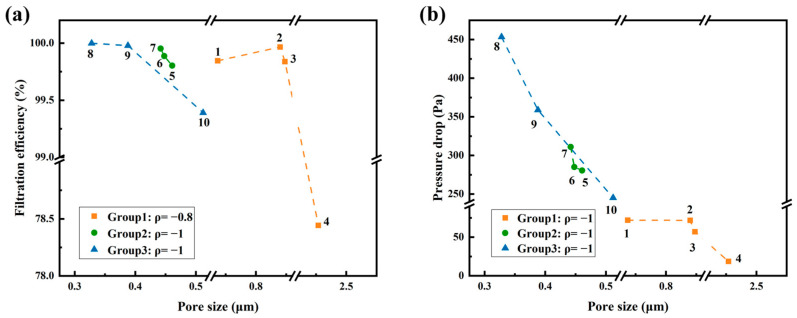
Relationships between pore size and filtration performance: (**a**) Pore size and filtration efficiency for Samples 1–10; (**b**) Pore size and pressure drop for Samples 1–10. The legend reports the within-group Spearman rank correlation coefficient (*ρ*).

Figure 9a,b further quantifies the relationships between pore size and filtration performance. In Figure 9a, all three groups show negative correlations between pore size and η. Group 1 exhibits a strong but not perfect negative trend (Spearman *ρ* = −0.8), while Groups 2 and 3 show nearly perfect negative monotonic relationships (*ρ* = −1), indicating that smaller pore sizes consistently yield higher filtration efficiency within each group. In Figure 9b, all groups show strong negative correlations between pore size and pressure drop, with *ρ* = −1, indicating that smaller pore size consistently led to higher ΔP. These trends reflect monotonic relationships under the tested structural and operating conditions. Due to the limited number of samples in Groups 2 and 3, the *ρ* values serve as indicative rather than statistically rigorous measures.

In summary, Figure 8 and Figure 9 collectively indicate that reducing pore size and distribution width enhances filtration efficiency but substantially increases pressure drop, resulting in an overall decline in QF. Therefore, when pursuing denser membrane structures, it is essential to balance efficiency gains with the corresponding increase in flow resistance. Optimal design should target the required efficiency while minimizing unnecessary increases in ΔP to maintain a favorable QF.

### 3.5. Hierarchical Linkage Between Material Parameters, Structure, and Filtration Performance of Biaxially Stretched PTFE Membrane

Based on the above analysis, a hierarchical linkage from material characteristics to fiber structure and ultimately to filtration performance can be established. At the material level, the Mn values in Table 1 and the particle size distributions in Figure 2 jointly influence fiber formation during stretching. While Mn varies significantly across samples, the average particle size and its CV remain relatively concentrated. Generally, a higher Mn combined with a lower particle size CV facilitates sufficient chain entanglement and more uniform strain distribution during stretching, although it does not solely determine the final fiber scale.

At the fiber structure level, Figure 4 shows a progressive narrowing of fiber diameter distributions across the three groups, and Figure 5 further reveals monotonic trends between Mn/particle size and fiber diameter within groups. Overall, samples with higher Mn and lower particle size CV tend to exhibit smaller average fiber diameters and lower CV. However, Sample 4 with extremely low Mn-suffered from inadequate chain entanglement, resulting in excessive node merging and fiber thickening during stretching and sintering. This disrupted the uniform fiber network and led to abnormally large pores. The anomalous behavior of Sample 4 underscores the critical importance of selecting resins with sufficiently high molecular weight to ensure adequate entanglement density and stretchability, which are essential for producing uniform and dense fibrous structures.

At the pore structure level, a uniform fiber network corresponds to finer and more evenly distributed pores. Figure 6 demonstrates that both the average pore size and distribution width decrease from Group 1 to Group 3. Figure 7 indicates that in Groups 2 and 3 where pore size CV are lower, a clearer positive correlation exists between average fiber diameter and pore size, with the former serving as a geometric predictor of inter-fiber spacing and pore dimensions.

Finally, these structural differences translate into filtration performance outcomes. Figure 8 and Figure 9 show that within each group, average pore size is negatively correlated with filtration efficiency and positively correlated with pressure drop. In general, finer and more uniform fibers, along with smaller and narrower pore distributions, contribute to higher filtration efficiency but also increase pressure drop, thereby reducing the QF. Sample 4 stands out as an outlier, where its anomalous pore structure significantly reduced efficiency and QF, weakening the monotonic fiber–pore–performance relationship within Group 1. Nonetheless, even when excluding Sample 4, the progressive trends observed across material, structure, and performance from Group 1 to Group 3 remain consistent, indicating the robustness of the established correlation chain.

### 3.6. Air Permeance Comparison Between PTFE Membranes and Commercial PP Electret Filter

**Figure 10:**
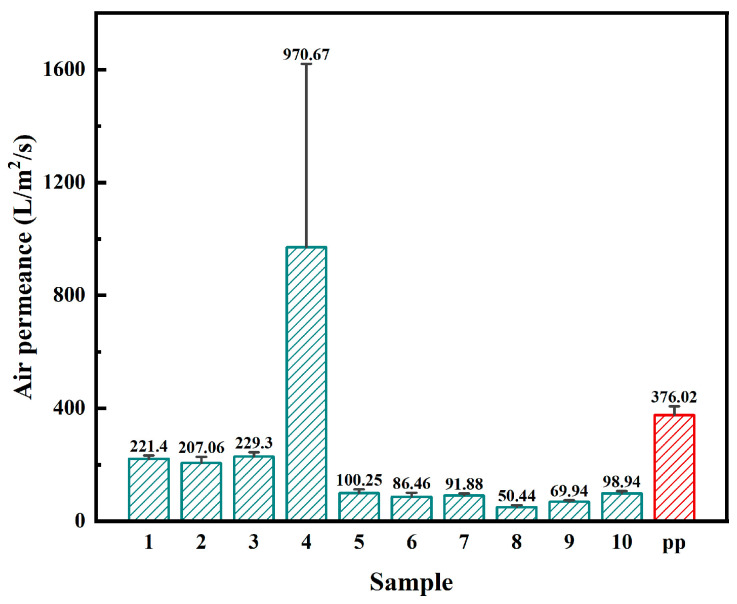
Air permeance of PTFE membranes and commercial PP electret filter. Green indicates PTFE, and red indicates PP.

Under identical test conditions, air permeance is a key indicator of ventilation performance and energy consumption for filter media. In this study, the air permeance of Samples 1–10 and a commercial PP electret filter was measured at a pressure differential ΔP = 200 Pa and an effective test area of 20 cm^2^, as shown in Figure 10. Excluding the structurally abnormal Sample 4, the biaxially stretched PTFE membranes exhibit air permeance values mainly in the range of about 50–230 L·m^−2^·s^−1^, whereas the commercial PP electret filter shows a significantly higher air permeance of 376 ± 31 L·m^−2^·s^−1^ under the same conditions. Owing to its enlarged mean pore size and broad pore-size distribution, Sample 4 reaches an air permeance of 971 ± 649 L·m^−2^·s^−1^, which is consistent with its lower filtration efficiency η and inferior QF discussed earlier. Overall, at the same pressure drop, the lower air permeance of the PTFE membranes (except Sample 4) compared with the PP electret filter reflects their finer and denser pore structures, in agreement with the pore-size and filtration analyses.

It should be emphasized that there is a typical engineering trade-off between air permeance and filtration efficiency: at a fixed pressure drop, higher air permeance usually implies lower efficiency, whereas improving efficiency inevitably leads to some increase in flow resistance. Commercial PP electret filter rely on stored electrostatic charges to capture particles and can initially achieve high efficiency at relatively low pressure drops; however, previous studies have shown that these charges can decay rapidly under high temperature, high humidity or organic vapor exposure, leading to a decline in filtration efficiency over service time [15,16,17]. In contrast, the PTFE membranes developed in this work operate via purely mechanical filtration through the node–fibril pore network, with particle capture dominated by diffusion and interception, and are essentially insensitive to environmental temperature and humidity or most organic vapors. Related studies have reported that PTFE-based filter materials can maintain stable efficiency and pressure drop even after multiple pulse-jet cleaning or washing cycles, demonstrating good regenerability and service-life stability [14,22,23].

From an application perspective, although the unit-area material cost of PTFE membranes is clearly higher than that of PP electret filters and societal concerns have been raised regarding environmental issues associated with certain fluoropolymer production routes involving per- and polyfluoroalkyl substances (PFAS), PTFE still offers distinctive advantages in scenarios where durability and stability are critical. In high-humidity, high-temperature or chemically aggressive exhaust streams (e.g., containing acids, bases or solvent vapors) in industrial, pharmaceutical and fine-chemical processes, PP electret filter may suffer from rapid charge decay and even matrix degradation, whereas PTFE membranes, owing to their excellent thermal and chemical resistance, can maintain stable filtration performance over extended service periods and can be regenerated multiple times by online or offline cleaning [22,23]. In recirculating filtration for semiconductor and flat-panel-display cleanrooms, heating, ventilation and air conditioning (HVAC) systems for automotive and rail transport, and high-grade HEPA and ULPA filters, where long replacement intervals and stable efficiency are required, PTFE membranes have the potential to offset their higher initial material cost by reducing replacement frequency and maintenance, and thereby to achieve a competitive or even favorable life-cycle cost [8,9,10].

In summary, the air permeance comparison indicates that, at the same pressure drop, the PTFE membranes prepared in this work exhibit higher flow resistance than the commercial PP electret filter, which is consistent with their finer pore structures and higher filtration efficiencies. For practical system design, it is therefore necessary to balance target filtration efficiency, allowable pressure drop, material cost, operating energy consumption and replacement interval. When long-term stability, environmental robustness and regenerability are required, PTFE membranes represent a promising candidate; in applications with lower demands on service life and stability and stronger sensitivity to initial material cost, PP electret filter remain a competitive choice.

## 4. Conclusions

This study systematically established a hierarchical correlation pathway linking material characteristics, structure, and performance for PTFE membranes based on resins with significantly varied Mn and particle size characteristics. The results show that high Mn and low particle size CV promote the formation of fine and uniform fiber networks during stretching, which subsequently leads to smaller and more concentrated pore structures and, in turn, significantly enhanced filtration efficiency. However, while reduced pore size strengthens diffusion and interception mechanisms, it also increases pressure drop and can diminish the QF, indicating that maximizing efficiency does not necessarily maximize QF and that an engineering trade-off between efficiency and resistance is required. Among all samples, Sample 8 represents the best overall compromise between high efficiency and acceptable resistance, with an Mn of 6.81 × 10^7^ g·mol^−1^, an average fiber diameter of 126.75 nm, a pore size of 0.328 μm, and a filtration efficiency of 99.9999%, together reflecting highly uniform structure and a favorable QF level within the sample set. In contrast, Sample 4 showed poor performance due to insufficient chain entanglement resulting from low Mn, which led to structural defects during fibrillation and sintering, a large average pore size of 2.454 μm, a much lower filtration efficiency of 78.442%, and a QF markedly below other samples in the same group. These findings underscore the critical importance of matching material parameters with processing conditions to ensure sufficient chain entanglement and a stable stretching window. Comparison with a commercial PP electret filter shows that, at the same pressure drop, the PTFE membranes in this study generally exhibit higher flow resistance (lower air permeance), which is consistent with their finer, denser pore structures and higher filtration efficiencies. Overall, the study establishes a hierarchical linkage from material parameters to structure and filtration performance, offering both theoretical and practical guidance for PTFE membrane material selection, structural design, and the development of high-performance air filtration media under realistic efficiency–resistance constraints.

## Figures and Tables

**Table 1:** Mn of PTFE resins, process parameters, and membrane thicknesses for preparing biaxially stretched PTFE membranes.

Samples	Mn (×10^7^ g∙mol^−1^)	Additive (%)	Inlet/Outlet Compression Ratio, (RR)	Machine-Direction Draw Ratio (×)	Transverse Draw Speed (m/s)	Pre-Stretched Thickness (µm)	Post-Stretched Thickness (µm)
1	4.92	26	83	12	6.45	260.0 ± 0.75	3.7 ± 0.011
2	2.38					278.8 ± 1.17	4.9 ± 0.021
3	1.84					266.8 ± 0.75	5.8 ± 0.016
4	1.13					291.2 ± 0.98	3.4 ± 0.011
5	11.9	28	83	10	5.5	335.8 ± 3.54	3.9 ± 0.041
6	5.67					342.2 ± 0.98	3.6 ± 0.010
7	2.81					318.6 ± 3.50	3.5 ± 0.038
8	6.81	28	60	10	3.08	320.0 ± 2.28	3.8 ± 0.027
9	5.21					322.2 ± 1.72	4.3 ± 0.023
10	3.94					317.4 ± 1.02	3.9 ± 0.013

## Data Availability

The original contributions presented in this study are included in the article. Further inquiries can be directed to the corresponding author.
